# Changes of the ecological environment status in villages under the background of traditional village preservation: a case study in Enshi Tujia and Miao Autonomous Prefecture

**DOI:** 10.1038/s41598-024-84101-z

**Published:** 2025-01-09

**Authors:** Fu Li, Huiqiong Xia, Jie Miao, Jialin Yang

**Affiliations:** Faculty of Resources and Environmental Science, Hubei University, Wuhan, China

**Keywords:** Ecological environment status, Traditional villages, Ecological index, Spatial–temporal change, Environmental impact, Environmental sciences, Environmental social sciences

## Abstract

The preservation of Chinese traditional villages plays a crucial role in promoting the sustainable development of rural natural, cultural, and ecological environments. It is also a key strategy for achieving rural revitalization. Current research on traditional villages predominantly focuses on the realm of cultural landscapes, with an emphasis on preserving the cultural ecological value of these communities. In comparison, discussions on the quality of the ecological environment of villages from the perspectives of natural environment, economic environment, and the social organizational environment within regional development are relatively scarce. Our study employed GIS and RS technology and refers to the Technical Criterion for Ecosystem Status Evaluation. Several sub-indices of the ecological environment status, including the biological richness index, vegetation coverage index, water network density index, and land stress index, were selected to construct an ecological environment assessment model. This model was used to analyze the spatial–temporal changes in the ecological environment status of each county, county-level city, and traditional village within the jurisdiction of Enshi Tujia and Miao Autonomous Prefecture and its surrounding areas from 2010 to 2020. The study quantitatively evaluated the ecological environment status of each county, county-level city, and village in Enshi before and after the implementation of traditional village preservation policies. Through comparative analysis, the study revealed the impact of these policies on the natural ecological environment of the study area. The results indicated the following: (1) From 2010 to 2020, the ecological index (*EI*) values in the villages of Enshi Prefecture exhibited a similar trend to the *EI* values in the respective counties and county-level cities they are located in, although significant differences in magnitude of change were observed. (2) The *EI* values in the counties, county-level cities, and villages demonstrated greater variation in the latter five years of the decade (2015–2020) compared to the previous five years (2010–2015). (3) In 2020, the *EI* value of the villages experienced more significant changes compared to 2010, whereas the overall *EI* value of the counties and county-level cities showed less pronounced changes. The findings of this study suggest that the traditional village preservation policies implemented in Enshi Prefecture have both positive and negative impacts on the ecological environment of the surrounding areas of protected villages, and these impacts become increasingly evident over time. By comparing and analyzing the ecological changes in the surrounding areas of traditional villages in Enshi Prefecture with the overall ecological changes in the respective counties and county-level cities, our study employs quantitative analytical methods to delve into the impact of traditional village conservation policies on the natural ecological environment. It assesses the effects of policy implementation on the natural ecological environment of traditional villages, analyzing both the positive and negative impacts brought about by the execution of these policies, with the aim of effectively guiding the natural ecological conditions of traditional villages towards a more healthy trajectory of development.

## Introduction

Before the 1980s, urbanization levels in China were relatively low, with an urbanization rate of only 20%^1^. The majority of the population resided in vast rural areas, adhering to traditional agricultural practices, and awareness of rural heritage preservation was still in its infancy. With the implementation and gradual advancement of the reform and opening-up policy, China experienced rapid economic growth, accompanied by the swift expansion of cities and the relative decline of certain rural areas. This shift placed numerous valuable historical and cultural assets—such as historical districts, heritage buildings, and renowned towns and villages—at risk of disappearance. Traditional villages, as crucial carriers of Chinese traditional culture, encapsulate both tangible and intangible heritage from the era of agrarian civilization^2^ and possess unique and irreplaceable attributes that are impossible to replicate. China has placed significant emphasis on the preservation of traditional villages, conducting and completing five nationwide surveys since 2012, resulting in the inclusion of 6819 villages in the official list of Chinese traditional villages. In recent years, with the implementation of the rural revitalization strategy, China has undertaken the adaptive reuse of traditional villages, injecting new vitality into these ancient settlements while ensuring their protection. However, the accelerated pace of urbanization has posed numerous challenges to traditional villages, including constructive destruction, ecological degradation, and inefficient use of land resources^3,4^. Additionally, economic development strategies in traditional villages—particularly tourism development—have led to changes in land-use patterns, degradation of agricultural ecosystems, soil quality decline, and pollution of water bodies, thereby introducing significant ecological risks^5^. Thus, the ecological environment protection of traditional villages has become an urgent issue that demands immediate attention.

In the *Guiding Opinions on Effectively Strengthening the Protection of Chinese Traditional Villages*, jointly issued in 2014 by the Ministry of Housing and Urban–Rural Development, the Ministry of Culture, and other departments, the protection of traditional villages was mandated to "emphasize the continuity of the ecological environment, respect the harmonious coexistence of people and nature, and strictly prohibit excessive development at the expense of the ecological environment" (https://www.mohurd.gov.cn/gongkai/zhengce/zhengcefilelib/201404/20140429_217798.html). Subsequently, the General Office of the State Council issued the *Opinions on Strengthening the Protection and Inheritance of Historical Culture in Urban and Rural Development* (https://www.gov.cn/gongbao/content/2021/content_5637945.htm), which incorporated traditional villages into the system of urban and rural historical and cultural protection. This policy aims to safeguard traditional villages’ spatial layout, historical appearance, cultural atmosphere, and natural landscapes, including topography, river, and lake systems, while emphasizing the preservation of traditional architectural wisdom to ensure holistic protection. However, at the present stage, the preservation of traditional villages focuses on tangible and intangible cultural heritage, and often neglects the ecosystem that carries such cultural heritage^6,7^.Consequently, many traditional villages with high ecological sensitivity and fragile ecosystems lack systematic protection, undermining the comprehensive implementation of traditional village preservation policies^8^. Additionally, as China intensifies its commitment to natural ecosystem and environmental protection, understanding the impact and mechanisms of traditional village preservation on the local ecological environment under the long-term implementation of these policies has become a crucial research question.

Current academic research on traditional villages primarily focuses on cultural landscapes, encompassing aspects such as the historical and cultural value of villages^9,10^, landscape patterns^11–13^, strategies for conservation and development^14,15^, the phenomenon of village hollowing^12,16^, spatial patterns and their evolutionary characteristics^17,18^, and environmental vulnerability^19^. In contrast, relatively few studies have examined the ecological impacts of traditional villages from the perspectives of the natural environment, economic environment, and social organizational context within regional development. Most research tends to focus on the cultural-ecological value of village preservation^20–22^. In the context of traditional village preservation, the dual core tasks of “protection” and “development” introduce complex effects on natural ecosystems: on one hand, protection measures help mitigate ecological degradation, while on the other, development activities—though fostering economic growth—inevitably trigger a range of ecological challenges^23^. Currently, research is limited regarding the ecological status of traditional villages under policy-driven conservation efforts. This study aims to explore the impacts of traditional village protection policies on the natural ecological environment of these villages, assessing the positive or negative effects of policy implementation (considering the double-edged effect of protection and development policies on natural ecosystems). Based on these insights, we further explore how policy measures can effectively guide the ecological status of traditional villages toward positive outcomes.

Ecological environment quality assessment is instrumental in gauging the current state of sustainable development within a region, thereby enabling the formulation of targeted ecological conservation strategies^24^. Based on the background of traditional village preservation and development policies, this paper constructed an ecological environment condition evaluation index system by taking the counties and cities of Enshi Prefecture and the areas surrounding traditional villages within the jurisdiction of each county and county-level city as two independent research areas, and developed an ecological environment evaluation index system from the aspects of the degree of vegetation coverage, the abundance of water, the degree of the land quality suffering from stress and the abundance and poverty of living organisms, etc., and through analyzing, evaluating and comparing the ecological environment condition and its sub-indices of both regions. It further proposes a scientific and effective scientific environment preservation perspective. By analyzing, evaluating and comparing the spatial and temporal characteristics of the ecological environment and its sub-indices, the study explores the extent and mechanism of the impact of the implementation of the traditional village preservation policy on the ecological environment of the surrounding areas, and further proposes scientific and effective recommendations for the preservation and development of traditional villages from the perspective of ecological environmental preservation, to achieve the harmony and unity of the development of the traditional villages in terms of cultural, economic and social development and the preservation of the natural ecological environment. The results of the study provide scientific guidance for the evaluation of the ecological environmental benefits under the traditional village preservation policy, and also provide some references for the formulation of traditional village preservation and development strategies in Enshi Prefecture.

## Material and methods

### Study region

**Fig. 1:**
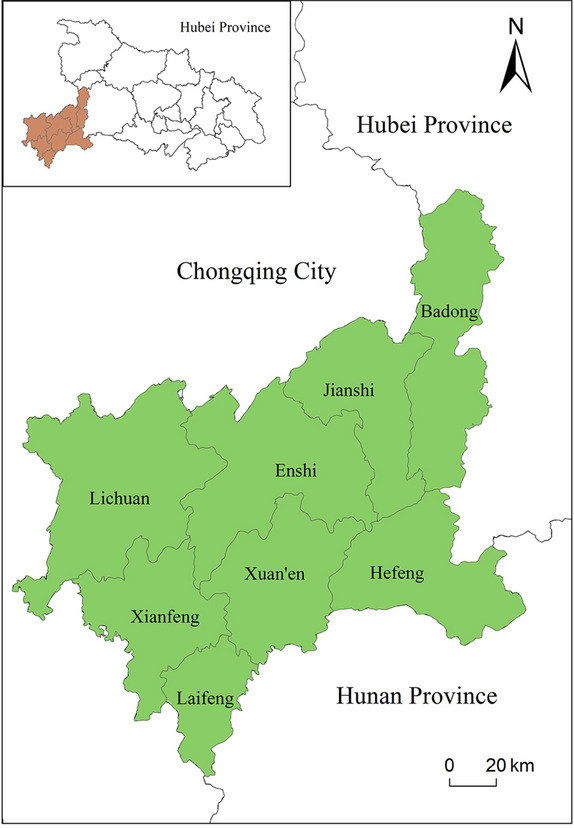
Enshi Tujia and Miao autonomous prefecture location map.

Enshi Tujia and Miao Autonomous Prefecture is located at the intersection of Hunan, Hubei and Chongqing provinces (cities) in China, at longitude 108° 23′ 12″ ~ 110° 38′ 08″ E, latitude 29° 07′ 10″ ~ 31° 24′ 13″ N.The state covers an area of 24,060.26 km^2^. The state belongs to the subtropical monsoon and monsoon humid climate, with abundant rainfall throughout the year. But with complex topography of different heights, the vertical geographical differences in climate are obvious. The territory is rich in water resources, and river networks are dense. The forest coverage is up to 74%, with a wide variety of biological species. It is an important "gene pool of plants and animals" in central China. Enshi Prefecture has 8 counties and county-level cities under its jurisdiction (Fig. 1), with a resident population of 3,456,100. From December 2012 to June 2020, the Ministry of Housing and Urban–Rural Development of the People’s Republic of China announced the first to five batches of Chinese traditional villages. Till 2020, A total of 206 villages were selected in Hubei Province, of which 81 villages were selected in Enshi Prefecture, accounting for 39.32% of the total number of villages in Hubei Province. We presented the number of traditional villages added in each county of Enshi Prefecture from 2010 to 2015 and from 2015 to 2020, as shown in Table 1.

**Table 1:** Changes in the number of traditional villages in counties and cities of Enshi Prefecture from the year 2010 to 2020.

	Enshi City	Lichuan City	Jianshi City	Badong County	Xuan’en County	Xianfeng County	Laifeng County	Hefeng County
2010 ~ 2015	3	9	1	0	4	5	10	5
2015 ~ 2020	9	9	1	1	11	3	6	4

### Data collection and processing

Remote sensing image datas of the study area were obtained from the NASA website (https://ladsweb.modaps.eosdis.nasa.gov/). MOD13A2 images were selected from May to September of 2010, 2015 and 2020. The NDVI annual data was generated after removing the negative values and maximum synthesis. The coordinate system was adopted as the WGS- 84 coordinate system and Albers projection. Land use data of the corresponding years were sourced from the China Multi-Period Land Use Remote Sensing Monitoring Dataset (CNLUCC) of the Center for Resource and Environmental Science and Data of the Chinese Academy of Sciences (https://www.resdc.cn/). Elevation data were retrieved from the global 30-m resolution of the National Aeronautics and Space Administration (NASA).DEM data (NASA DEM) were converted to 1 km × 1 km raster by projection transformation and resampling. Total water resources were obtained from Enshi Water Resources Bulletin, and the lengths of the rivers were derived from the above DEM data by hydrological analysis. The traditional villages data were taken from the "List of Villages in the First to Fifth Batches of Chinese Traditional Villages Inventory of the Ministry of Housing and Urban–Rural Development of the People’s Republic of China". The addresses of the traditional villages were converted into geographic coordinates after spatialization and were imported into ArcGIS to generate point data Buffer zone analysis was used to obtain the range of impacts of human activities and other factors on the ecological environment under the preservation policy of traditional villages.

### Methods

In our research, we refer to the work of Zeng et al.^8^, who evaluated the ecological environment of traditional villages in Dalu Village, Qinzhou, Guangxi. Their research combined land use survey data and official statistics, following technical standards for ecological environment assessment. However, their work was limited to assessing the ecological quality of a single village. Given our objective of exploring the impact of traditional village preservation policies on the surrounding ecological environment, we considered the overall changes in the ecological conditions of all traditional villages in Hubei Province.

### Buffer analysis

Existing research suggests that the primary activity areas of rural residents in China are concentrated within a 2–3 km radius around their residences, particularly in farmland and its surrounding areas^25–27^. This pattern is largely attributed to the fact that local residents’ work areas are typically within a reachable distance on foot or via short-distance transport^28^. Based on the concept of a "15-min living circle," relevant literature indicates that the average walking speed is around 4–5 km per hour, allowing a person to cover approximately 1000 to 1200 m in 15 min^29^. With economic development in rural areas, motorcycles and small farm vehicles have become common, with an average speed of no more than 20 km per hour in mountainous areas, expanding the 15-min activity range to around 5 km. Following tourism development in some traditional villages, villagers’ production and lifestyle patterns underwent significant changes, including adjustments to agricultural structures, alterations in cultivation practices, shifts in land use, and modifications in water resource utilization^27,30,31^. These changes have had varying degrees of impact on the surrounding natural environment. Given that the activity range for villagers’ production and daily life is approximately 5 km, this study defined an ecological environment zone for traditional villages as the area within a 5-km radius centered on each village.

Buffer analysis, a fundamental component of GIS spatial analysis, serves as an effective means to address issues of spatial proximity by describing the impact of geographic objects on surrounding areas^32^. Based on this, we developed a model centered on traditional villages, constructing buffers for 15-min, 30-min, and 45-min accessibility zones, corresponding to activity radii of 5 km, 10 km, and 15 km, respectively.

**Fig. 2:**
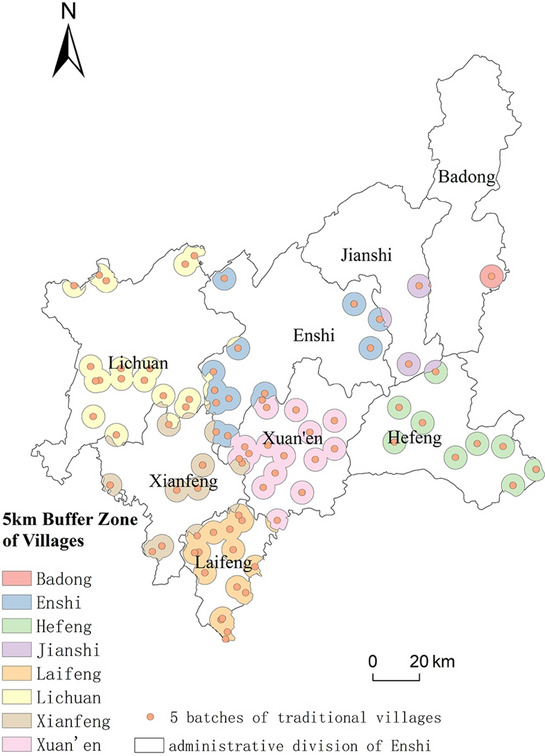
The 5KM activity range of villagers in traditional villages represented by buffer zones.

Using Geographic Information System (GIS) technology, we extracted the names of all villages listed in the national registry of traditional villages since 2010, converting them to specific latitude and longitude coordinates and projecting them into the WGS84 coordinate system. Using this data, we created 5-km, 10-km, and 15-km buffers for each traditional village in ArcGIS to evaluate the composite ecological environment quality indicators within these areas. Subsequently, we conducted an overlay analysis with the administrative layers of Hubei Province to calculate the average ecological environment index for traditional villages within each county or city and compared it with the overall ecological environment index of these administrative areas. We observed that the ecological environment quality in the 10- and 15-km buffers was not significantly different from that of the surrounding county or city. This suggests that, from the perspective of agricultural activities, the environmental impact of villagers’ daily activities is primarily concentrated within 5 km. This aligns with the "15-min living circle" concept found in the literature, so we used a 5-km buffer in subsequent analyses, assuming this as the maximum range within which human activity impacts the ecological environment of traditional villages (Fig. 2).

To better analyze the spatial and temporal distribution characteristics and differences of the ecological environment status of traditional villages within the counties and county-level cities in the study area, this paper took the surface data of a certain range of buffer zones around the traditional villages as the research unit for the evaluation of the ecological environment status of the villages. As the basic content of GIS spatial analysis, buffer zone analysis is an effective means to describe the influence of geographic objects on the surrounding area and solve the problem of spatial proximity. Concerning the methods of delineating village boundaries in the existing studies, combining with the actual situation of the study area, and taking into account the area and distribution of traditional villages in Enshi Prefecture, we have compared the results of the analysis of different buffer zones, and finally determined 5 km surrounding range of villages as the impact range of human activities under the preservation policy of traditional villages. The buffer zones of traditional villages in each county and county-level city in Enshi Prefecture were trimmed as the boundary, and the buffer zones of traditional villages in each county and county-level city were merged to obtain the buffer zone data of traditional villages in each county and county-level city (Fig. 2). The merged buffer zones of traditional villages in each county and county-level city could be regarded as a separate research unit as the surrounding range of traditional villages within the jurisdiction of each county and county-level city (hereinafter referred to as “villages”). This study carried out the spatial and temporal comparative analysis of ecological conditions based on the counties and county-level cities as well as the villages within the jurisdiction of them.

### Method for establishing an ecological environment assessment model

To further quantify changes in the ecological environment quality of traditional villages and their administrative areas, we selected the years 2010, 2015, and 2020 to calculate the ecological environment index (Ecological Index, *EI*) for traditional villages and their respective counties or cities within the study area. We then analyzed changes over the two five-year periods from 2010 to 2015 and 2015 to 2020, as well as the overall decade from 2010 to 2020. By comparing changes across different time spans, we aimed to determine whether shifts in the ecological quality around traditional villages were consistent with the general ecological quality trends in their counties or cities. If significant differences were observed, this would suggest that changes in the ecological environment around villages possess unique characteristics, potentially reflecting the specific impact of traditional village preservation policies on the surrounding environment. Through these methods, this study examined whether traditional village preservation policies impact the ecological environment of villages and further analyzes the specific trends and characteristics of changes in the ecological environment around these villages over time.

Evaluation of ecological environment status is to use *EI* to reflect the overall status of regional ecological environment. This paper refered to the Technical Criterion for Ecosystem Evaluation issued by the Ministry of Environmental Preservation of the State^33^, and selected four indices including the vegetation coverage index, the water network denseness index, the land stress index, and the biological richness index and get the normalized ecological environment status index of the study area. These indices are calculated as follows:

#### Vegetation coverage index (VCI)

1 $$VCI = NDVI = A_{veg} \times \left( {\frac{{\mathop \sum \nolimits_{i = 1}^{n} P_{i} }}{n}} \right)$$

where ${P}_{i}$ is the mean value of the monthly maximum value of *NDVI* (normalized vegetation index) from May to September. The *NDVI* data were obtained by remotely sensed data of MOD13A2 after removing the negative values and synthesizing maximum values. ${A}_{veg}$ is the normalization coefficient of the *VCI*.

#### Water network denseness index (WNDI)

2 $$WNDI = \, \left( {A_{riv} \times L_{r} /S + A_{riv} \times S_{w} /S + A_{res} \times Q_{w} /S} \right)/{3}$$

where *S* is the area of the region.* A*_*riv*_ is the normalization coefficient of the length of the river, with a reference value of 84.3704083981.* L*_*r*_ is the length of the river.* A*_*riv*_ is the normalization coefficient of the area of the watershed, with a reference value of 591.7908642005.* S*_*w*_ is the area of the watershed.* A*_*res*_ is the normalization coefficient of the amount of water resources, with a reference value of 86.3869548281.* Q*_*w*_ is the amount of water resources.

#### Land stress index (LSI)

3 $$LSI = A_{ero} \times \left( {0.{4} \times HEA + 0.{2} \times MEA + 0.{2} \times CA + 0.{2} \times OLS} \right)/S$$

where *S* is the area of the region. *A*_*ero*_ is the normalization coefficient of *LSI*, with a reference value of 236.0435677948. The mode of soil erosion in the study area is mainly hydraulic erosion. The degree of soil erosion in the study area is classified^34^ according to the "Soil Erosion Classification and Grading Standard"^35^. *HEA* is the area of eroded land. *MEA* is the area of moderately eroded land. *CA* is the area of construction land. *OLS* is other land stress.

#### Biological richness index (BRI)

4 $$BRI = A_{bio} \times \left( {0.{35} \times RA + 0.{21} \times GA + 0.{28} \times WA + 0.{11} \times CLA + 0.0{4} \times CA + 0.0{1} \times ULA} \right)/{\text{S}}$$

where *S* is the area of the region.* A*_*bio*_ is the normalization coefficient of the *BRI* with a reference value of 511.2642131067. *RA* is the area of forest. *GA* is the area of grass. *WA* is the area of water. *CLA* is the area of cropland. *CA* is the area of construction land. *ULA* is the area of unused land.

#### Ecological index (EI)

5 $$EI = 0.{25} \times VCI + 0.{2} \times WNDI + 0.{2} \times \left( {{1} - LSI} \right) + 0.{25} \times BRI$$

According to the Technical Criterion for Ecosystem Evaluation^33^ issued by the Ministry of Environmental Preservation of the State, *EI* is categorized into five levels according to the magnitude of the value including excellent, good, average, fair and poor, as shown in Table 2. Excellent means the region has high vegetation cover, rich biodiversity and stable ecosystems. Good indicates the area has high vegetation cover, rich biodiversity, and is suitable for human life. Average illustrates medium vegetation cover, average level of biodiversity in the region, which is more suitable for human life, but with the presence of constraints it’s not suitable for human life. If the status is fair, it means poor vegetation cover, severe drought, low rainfall and fewer species in the area, and there is presence of factors that clearly constrain human life. Poor illustrates conditions are relatively harsh, with constraints on human life.

**Table 2:** Grading of ecological status.

Level	Excellent	Good	Average	Fair	Poor
Value	*EI* ≥ 75	55 ≤ *EI* < 75	35 ≤ *EI* < 55	20 ≤ *EI* < 35	*EI* < 20

To assess the trends in ecological environment change, we conducted a quantitative analysis of the ecological environment index change values for each county and traditional village within the study area across two time periods: 2010–2015 and 2015–2020, the change value was represented by *ΔEI*. All change values were classified into four levels using the natural breaks classification method: no apparent change, slight change, apparent change, and significant change. If there was no significant change in ecological environment quality during the specified period, the change value was close to zero; conversely, a non-zero change value indicated a change in environmental quality. We used three levels—slight change, apparent change, and significant change—to quantify the degree of ecological environment quality change. Notably, a negative change value indicates deterioration in environmental quality, while a positive value indicates improvement. Detailed classification results are presented in Table 3.

**Table 3:** Grading of the degree of change in ecological status.

Level	No apparent changes	Slight changes	Apparent changes	Significant changes
Value change	|*ΔEI*|< 1	1 ≤|*ΔEI*|< 3	3 ≤|*ΔEI*|< 8	|*ΔEI*|≥ 8

## Results

According to the calculation method of the above evaluation indices, after normalizing we obtained the vegetation coverage index, water network denseness index, land stress index, biological richness index and ecological index for the three periods of 2010, 2015, and 2020 of counties and county-level cities as well as villages in Enshi Prefecture, respectively.

### Single-factor analysis of the status of the ecosystem

#### Vegetation coverage index

Based on the normalized vegetation index NDVI data for the three periods of 2010, 2015 and 2020, the vegetation coverage index for each period in the study area was calculated. The change of vegetation coverage index and spatial visualization in counties and county-level cities as well as villages in Enshi Prefecture are shown as the ten-year period from 2010 to 2020 (Fig. 4).

**Fig. 3:**
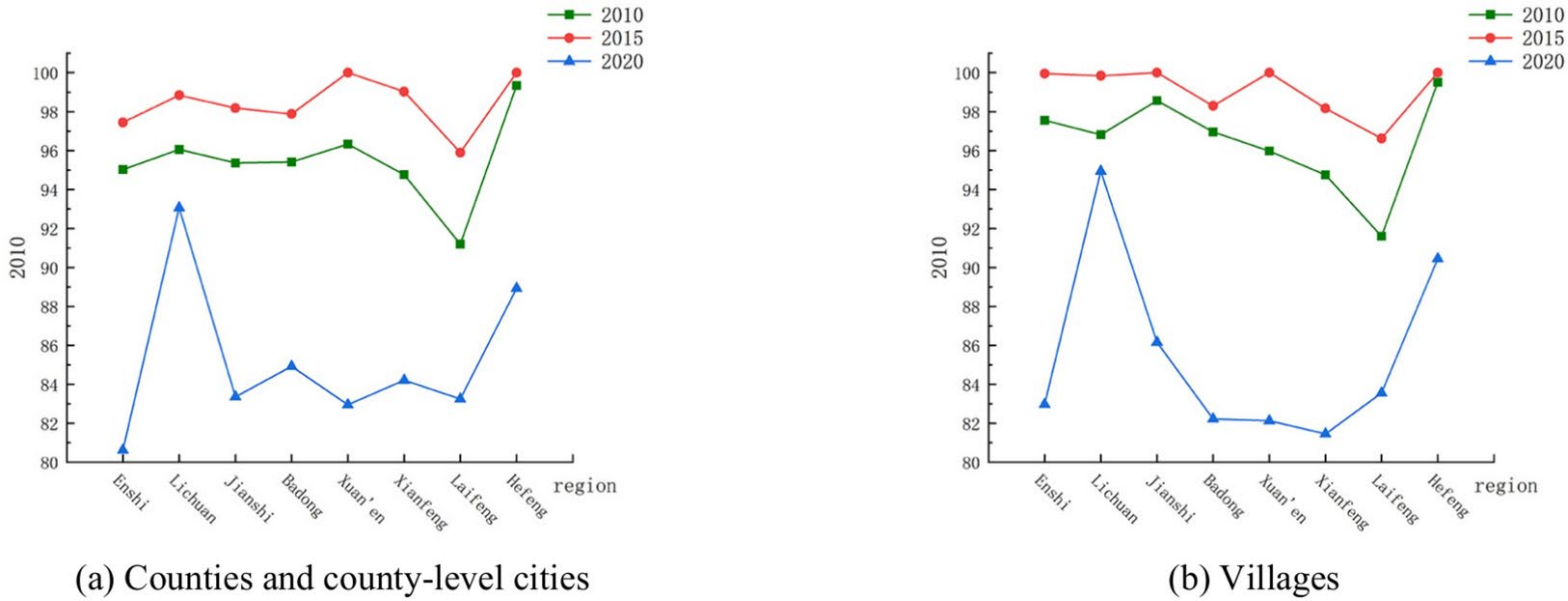
Changes inVCI of Enshi Prefecture from 2010 to 2020.

From Fig. 3a,b, it can be seen that in 2010 and 2015, except for Laifeng County, the vegetation coverage index values of all counties and county-level cities were above 95, and the differences were not significant. However, in 2020, the vegetation coverage indices of all counties and county-level cities declined, and the degree of the decline was significantly different. The vegetation coverage index values of villages in all counties and cities were somewhat different from those of their respective counties and cities, but as a whole, the distribution characteristics of the index values were relatively similar. The distribution characteristics of the index values were similar to those of the counties and county-level cities in which they were located. From the time point of view, the change trend of the vegetation coverage index of counties and county-level cities and villages in Enshi Prefecture between 2010 and 2020 was roughly close to each other, and in general, they all showed the trend of slightly increasing first and then decreasing. The vegetation coverage index of counties, county-level cities as well as villages was the highest in 2015, and the lowest in 2020, and there was a significant decrease in the vegetation coverage in 2020 compared with that in 2010 and 2015. After comparative analysis with the land use data changes in Enshi Prefecture, it was speculated that the decrease in the vegetation coverage index in 2020 may be related to the increase in the area of construction land. In terms of the degree of change, the change value of the vegetation coverage index of counties, county-level cities as well as villages between 2015 and 2020 was generally larger than that between 2010 and 2015, indicating that the change of vegetation coverage in the latter five years from 2010 to 2020 was more obvious.

**Fig. 4:**
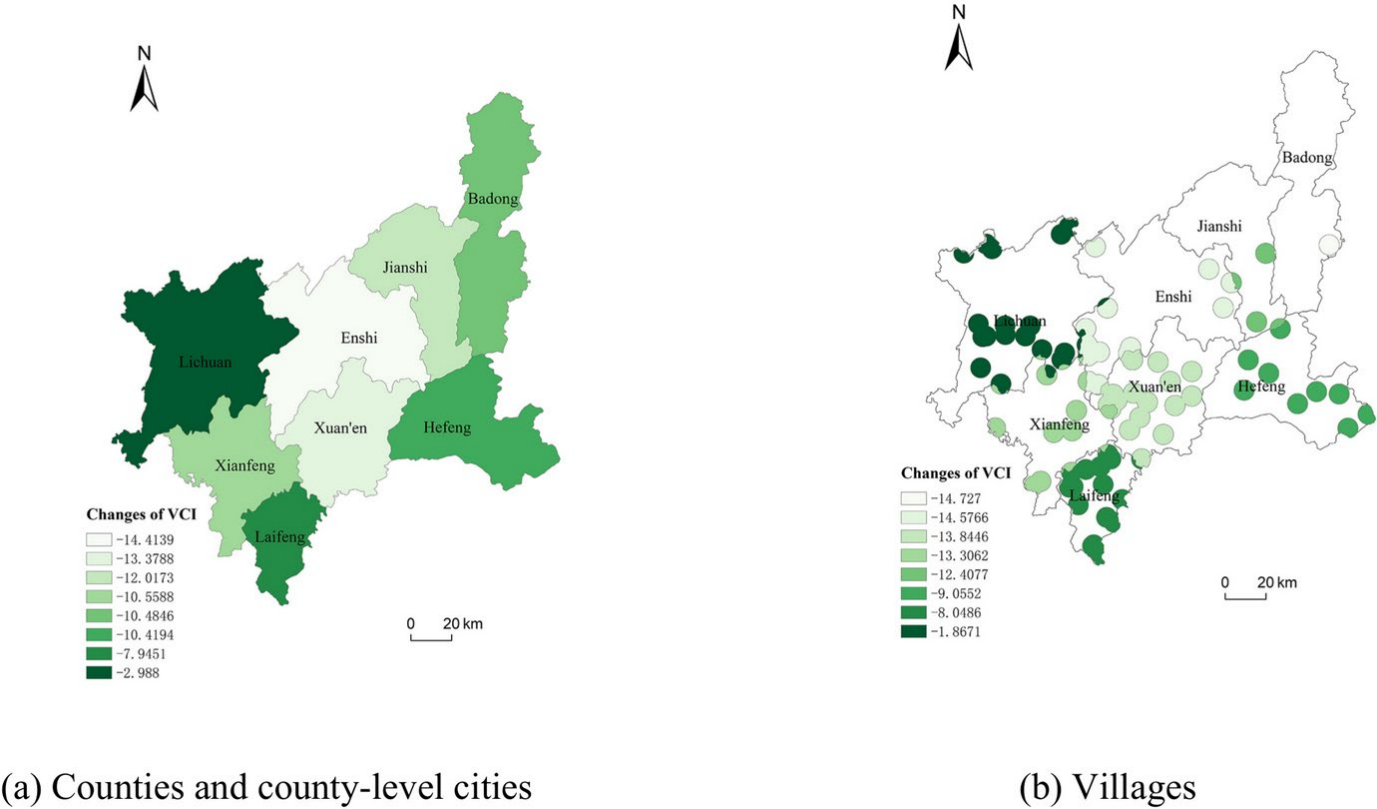
Spatial distribution of changes in VCI in Enshi Prefecture from 2010 to 2020.

In terms of spatial distribution (Fig. 4a), in 2020 compared with 2010, the vegetation coverage index changed more in the central part of the study area than in the neighboring regions. Among them, the vegetation coverage index of Enshi City decreased the most, with a value of 14.4139. Lichuan City decreased the least, with a value of 2.9879.The changes in the vegetation coverage index of villages were basically the same as that of the counties and county-level cities in which they were located, with the vegetation coverage index value of villages in Enshi City decreasing the most, with a value of 14.5766, and that of Lichuan City decreasing the least, with a value of 1.8671 (Fig. 4b).

#### Water network denseness index

**Fig. 5:**
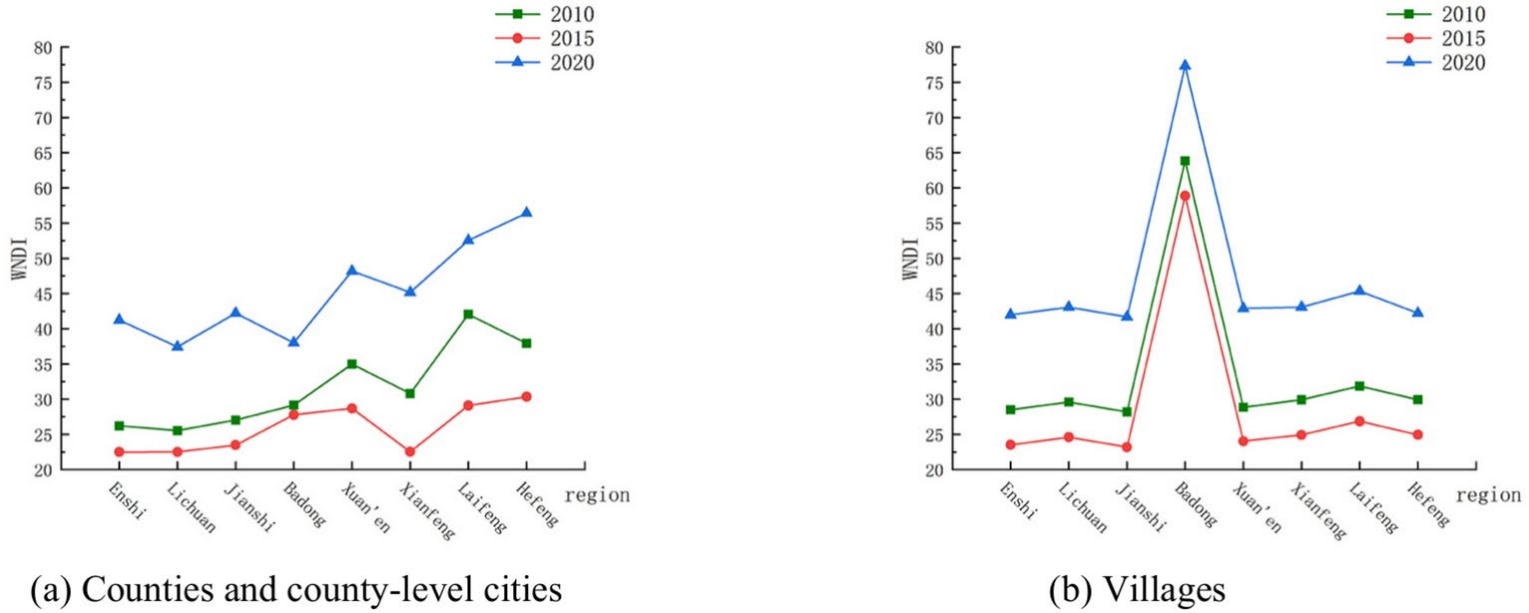
Changes in *WNDI* of Enshi Prefecture from 2010 to 2020.

From Fig. 5a,b, it can be seen that the water network denseness values of the counties and county-level cities in the study area differ significantly, with Xuan’en, Laifeng, and Hefeng counties having larger water network denseness than the other cities, and the water network denseness values of the villages, except Badong County, were relatively close to each other, the differences were smaller than those of the corresponding counties and county-level cities. In 2010 and 2015, the water network denseness values of the counties and county-level cities in the study area and the villages under their jurisdiction were generally low, while in 2020 the values of water network denseness increased significantly, and the water resources situation improved.

**Fig. 6:**
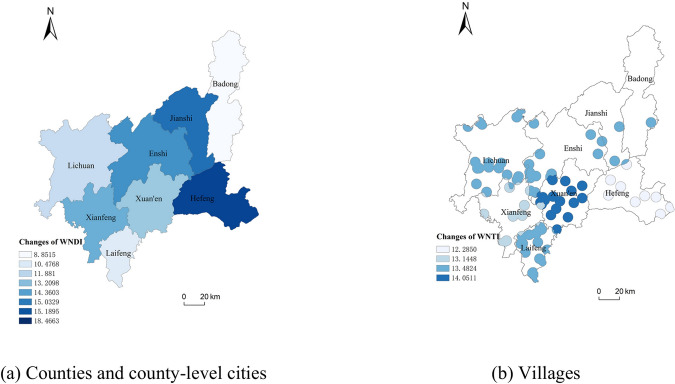
Spatial distribution of changes in WNDI in Enshi Prefecture from 2010 to 2020.

From the trend of water network denseness index values in different years in Fig. 5a,b, the water network denseness index of all counties and county-level cities of Enshi Tujia and Miao Autonomous Prefecture and the villages under their jurisdiction showed a slight decrease and then an increase from 2010 to 2020, in which the change of the water network denseness index from 2015 to 2020 was more obvious than that from 2010 to 2015. The comparison of water network denseness index values of the counties, county-level cities as well as villages under their jurisdiction found that the difference between Badong County and villages under its jurisdiction from 2010 to 2020 was larger, and the difference between other counties and county-level cities was smaller. Comparing the water network denseness index values of counties and county-level cities and the villages under their jurisdiction, it is found that the difference between the water network denseness values of Badong County and the villages under its jurisdiction between 2010 and 2020 was large, while other counties and county-level cities were smaller. From the spatial distribution of the water network denseness index changes in the counties and county-level cities in the study area in Fig. 6, the water network denseness index values in the east-central part of the study area changed significantly in 2020 compared to 2010, with Hefeng County rising the most significantly, with a value of 18.4663, and Badong County rising the least, with a value of 8.8515.Since the water network denseness index was related to the length of rivers, the area of watersheds, and the amount of water resources, etc., the water network denseness index of each village in Enshi Prefecture between 2010 and 2020 was found to have a large difference in value between villages under its jurisdiction. From 2010 to 2020, the river length and water area of each county and county-level city in Enshi Prefecture basically remained unchanged, and the water resources of Enshi Prefecture decreased by 41.737 × 109 m^3^ in 2015 compared with 2010, and increased by 154. 4995 × 109 m^3^ in 2020 compared with 2015, which was a big change, resulting in the obvious change of the water network denseness index.

#### Land stress index

**Fig. 7:**
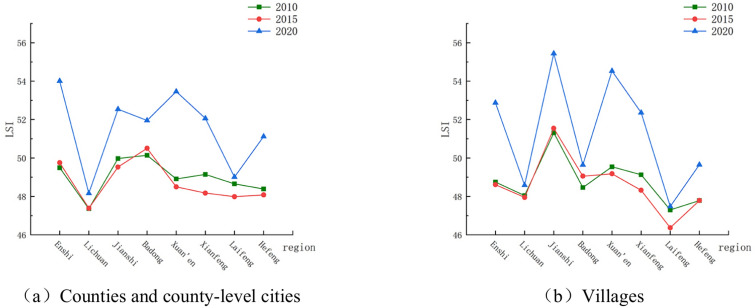
Changes in LSI of Enshi Prefecture from 2010 to 2020.

The land stress index of each county and county-level city in Enshi Prefecture and their jurisdictional villages from 2010 to 2020 is shown in Fig. 7. From Fig. 7a, it can be seen that in 2020 compared to 2010, the land stress index changes in all counties and county-level cities of Enshi Tujia and Miao Autonomous Prefecture were positive. The land stress index represents the degree of land stress, and the positive value of the change indicates that the degree of land stress has increased. From the land stress index curves of 2010, 2015 and 2020 in Fig. 7b, the land stress situation of counties and county-level cities in the study area and their jurisdictional villages remained almost unchanged in 2010 and 2015, and in 2020, except for Lichuan and Laifeng counties. All the other counties and county-level cities had different degrees of land stress, while the land stress situation of the villages, except for Badong County, was roughly the land stress situation of villages, except for Badong County, was roughly consistent with the situation in their counties and county-level cities. In terms of the degree of change, the change value of the land stress index between 2015 and 2020 in all counties and county-level cities was greater than that between 2010 and 2015. From 2010 to 2015, the change value of the land stress index was negative in all counties and county-level cities except for Enshi, Lichuan, and Badong counties, where the change value was positive. The change value in all counties and county-level cities from 2015 to 2020 was positive. In general, for all counties and county-level cities, during the ten years from 2010 to 2020, the land stress index in Xuanen County increased the most, 4.5514, with the greatest change in the degree of land under stress. The land stress index in Lichuan City increased the least, 0.7986, with the smallest change in the degree of land under stress. The land stress index of villages within the jurisdiction of all counties and county-level cities showed an increasing trend, with the greatest increase in Xuanen County, with an increase in the value of 4.9904 in Xuan’en County and 0.185225 in Laifeng County. There were obvious differences in the land stress indices of counties and county-level cities and villages under their jurisdictions over the past ten years, and the trends of changes were also different.

**Fig. 8:**
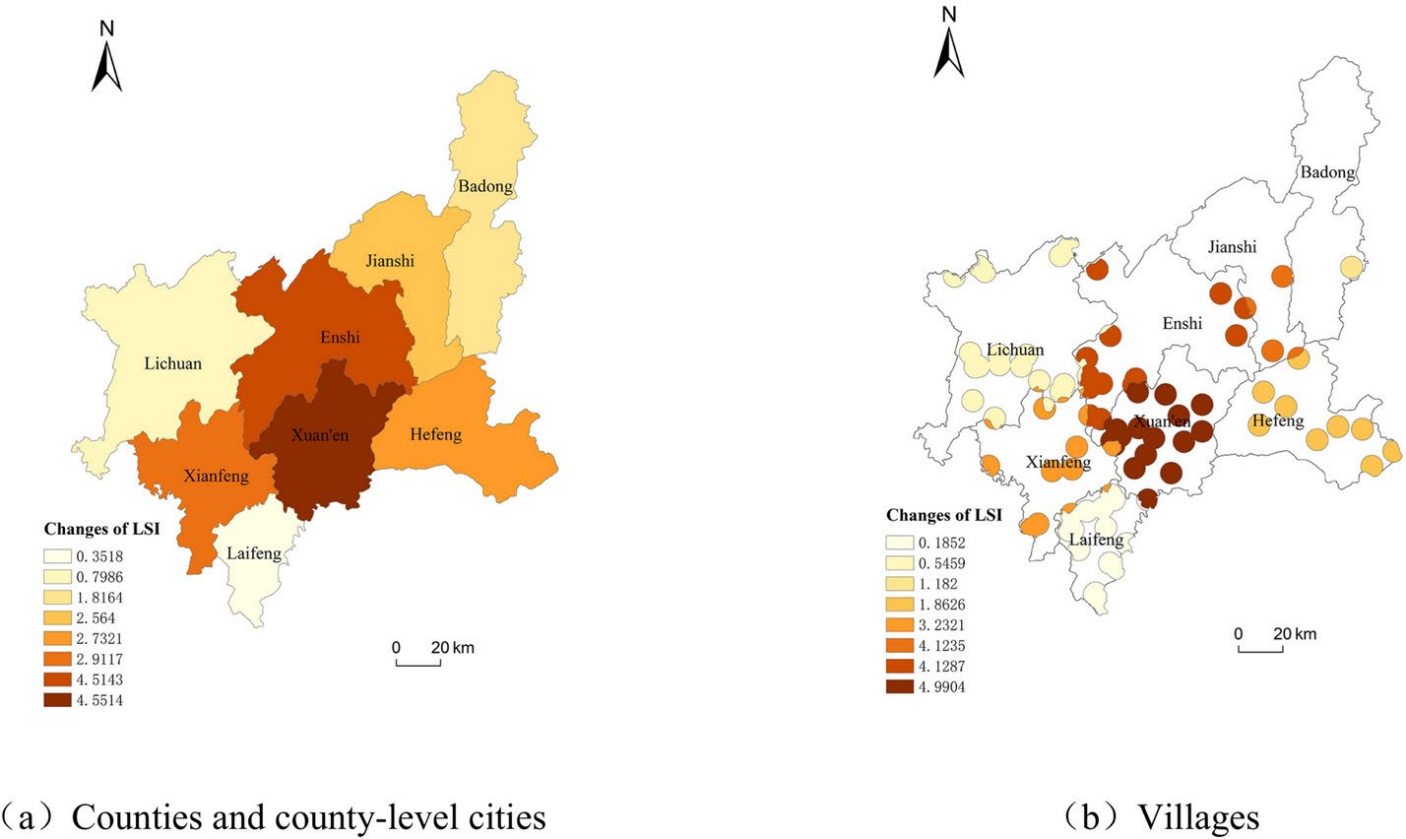
Spatial distribution of changes in LSI in Enshi Prefecture from 2010 to 2020.

Comparing the degree of soil erosion in Enshi Prefecture in 2010 and 2020, it can be found that soil erosion in Xuanen County is more serious (Fig. 8), the area of heavy erosion increased by 9.24%, and the degree of land under stress increased. This may be related to the increase of local construction land.

#### Biological richness index

**Fig. 9:**
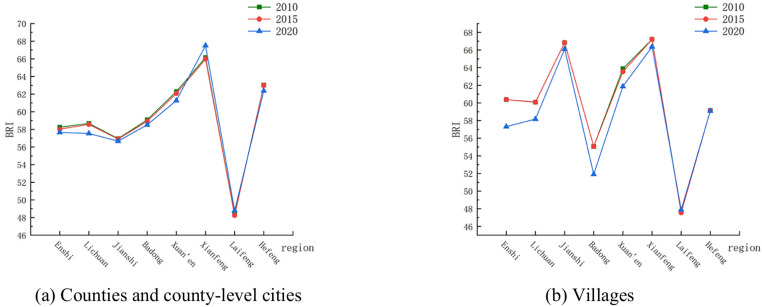
Changes in BRI of Enshi Prefecture from 2010 to 2020.

**Fig. 10:**
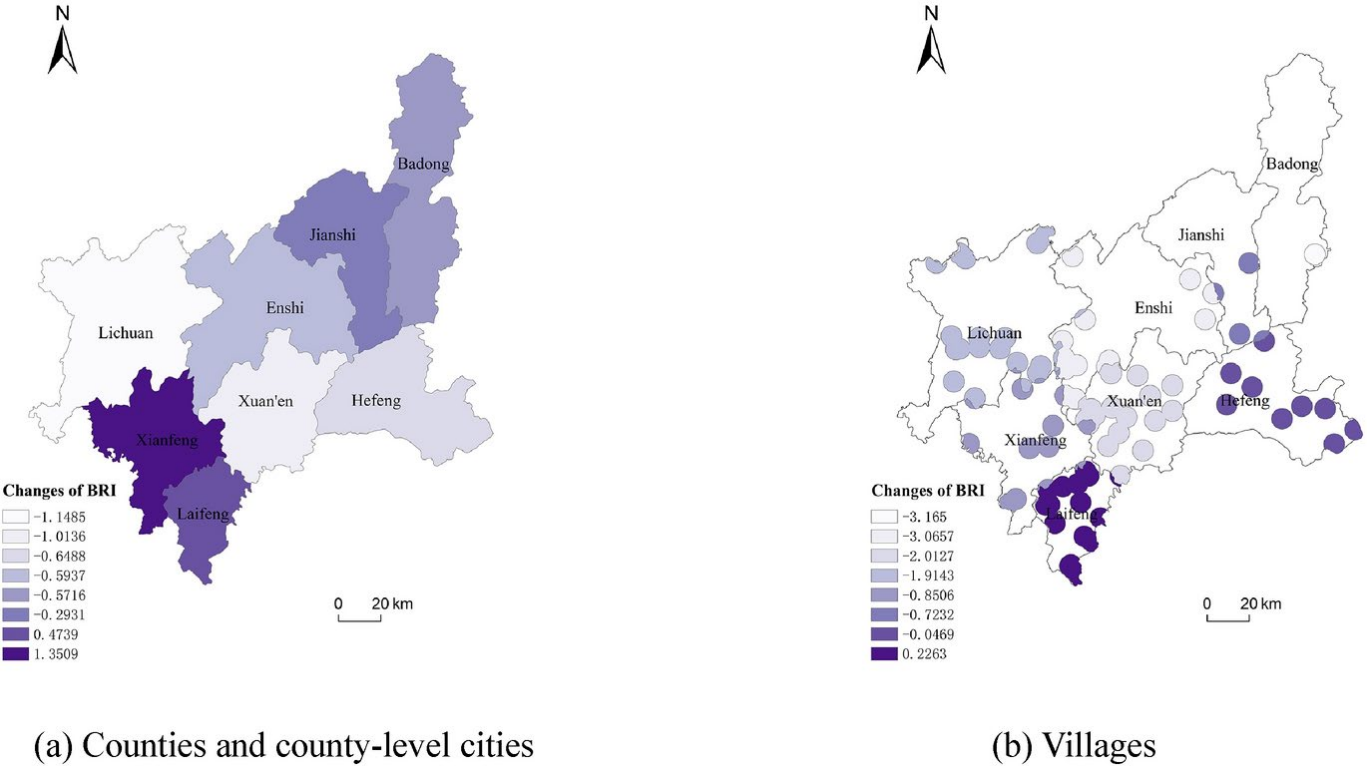
Spatial distribution of changes in BRI in Enshi Prefecture from 2010 to 2020.

Comparing the shapes of the curves in Fig. 9a,b, it can be seen that the curves of the habitat quality index of villages in counties and county-level cities and their jurisdictions in Enshi Prefecture vary greatly: during the ten years from 2010 to 2020, the change in the habitat index of counties and county-level cities in Enshi Prefecture was not obvious, while the change in the habitat quality index of villages in a few jurisdictions was greater, such as Enshi County, Lichuan County, Badong and Xuan’en Counties, and the magnitude of change in the habitat quality index of villages was greater than that of the counties and county-level cities. Most of the areas in Enshi Prefecture (except Xianfeng and Laifeng counties) had negative changes in the habitat quality index, indicating a general decline in biological abundance within the study area. The change in the habitat quality index between 2015 and 2020 was greater than that between 2010 and 2015 in all counties and county-level cities, and the change in value was mainly due to changes in the area of woodland and grassland. The habitat quality index of villages within the jurisdiction of each county and county-level city was negative except for Laifeng County (Fig. 9b), and the habitat conditions became worse. From the spatial distribution of changes in the habitat quality index (Fig. 10), the areas with improved habitat quality conditions in the decade from 2010 to 2020 are located in the western part of Enshi Prefecture, such as Xianfeng and Laifeng counties. Lichuan and Xuanen counties showed little change in the values of the habitat quality index, but the values were negative, which implied that the habitat quality conditions in these areas are deteriorating, and need to be paid attention to.

#### Analysis of the ecological condition

As can be seen from the grading of the ecological environment status (Table 4), the ecological environment status of all counties and county-level cities was at the level of “good” in the 10 years from 2010 to 2020, which indicated that the ecological environment status of Enshi Prefecture is relatively good, and the ecological environment was favorable to people’s production and life.

**Table 4:** *EI* of Enshi Prefecture in the year 2010, 2015 and 2020.

	Counties and city-level counties			Villages		
	2010	2015	2020	2010	2015	2020
Enshi City	59.4877	59.2184	57.7801	61.4739	61.1014	58.6275
Lichuan City	60.1863	60.2325	61.2538	61.5443	61.3233	62.9948
Jianshi City	59.1865	59.2538	58.6047	63.4104	62.7235	61.9272
Badong County	60.3318	60.5448	58.9176	66.5906	65.8085	64.2612
Xuan’en County	63.0983	62.7671	61.1306	62.2145	62.2123	59.8610
Xianfeng County	63.1755	62.7231	63.2983	63.3692	63.3863	61.7275
Laifeng County	58.3883	57.0837	58.592	56.4881	56.8950	57.2146
Hefeng County	64.8006	63.5032	65.1155	62.0043	61.1305	61.8085

By comparing the *EI* values of the counties and county-level cities in Enshi Prefecture at three points in time over ten years, it was found that there was little change in the ecological environment status between 2010 and 2015. Between 2015 and 2020 the change in the ecological environment status was more pronounced in the majority of regions, with more than half of the counties and county-level cities experiencing changes of about 2.5 percent. Among them, Enshi, Jianshi, Badong and Xuan’en saw a slight decrease in the ecological environment status index. Accordingly, the ecological condition of villages within the jurisdiction of counties and county-level cities did not change significantly in the first five years. In the second five years, the ecological condition was similar to that of the counties and county-level cities in which they are located, with a trend of more pronounced changes, of which most villages changed by more than 2.5%, and the areas around individual villages changed by 4%, while the ecological condition indices of Lichuan, Laifeng and Hefeng increased slightly. All counties and county-level cities and villages in all counties and county-level cities showed the phenomenon that the ecological and environmental conditions had become better in some areas and worse in others.

In comparison with the grading of the degree of change of the ecological environment status (Table 3), during the ten years, the ecological environment status of Enshi City, Lichuan City, Badong County and Xuan’en County changed slightly, and the rest of the counties and municipalities did not have any significant changes. The ecological environment status of Lichuan City has slightly changed for the better, and the ecological environment status of Enshi City, Badong County and Xuanen County has slightly changed for the worse. The *EI* values of villages in Enshi City, Lichuan City, Jianshi City, Badong County, Xuanen County and Xianfeng County changed slightly, and there was no significant change in the villages in Laifeng County and Hefeng County. Among them, the ecological condition of the villages under Lichuan City became slightly better, and Enshi City, Jianshi City, Badong County, Xuanen County and Xianfeng County became slightly worse.

**Fig. 11:**
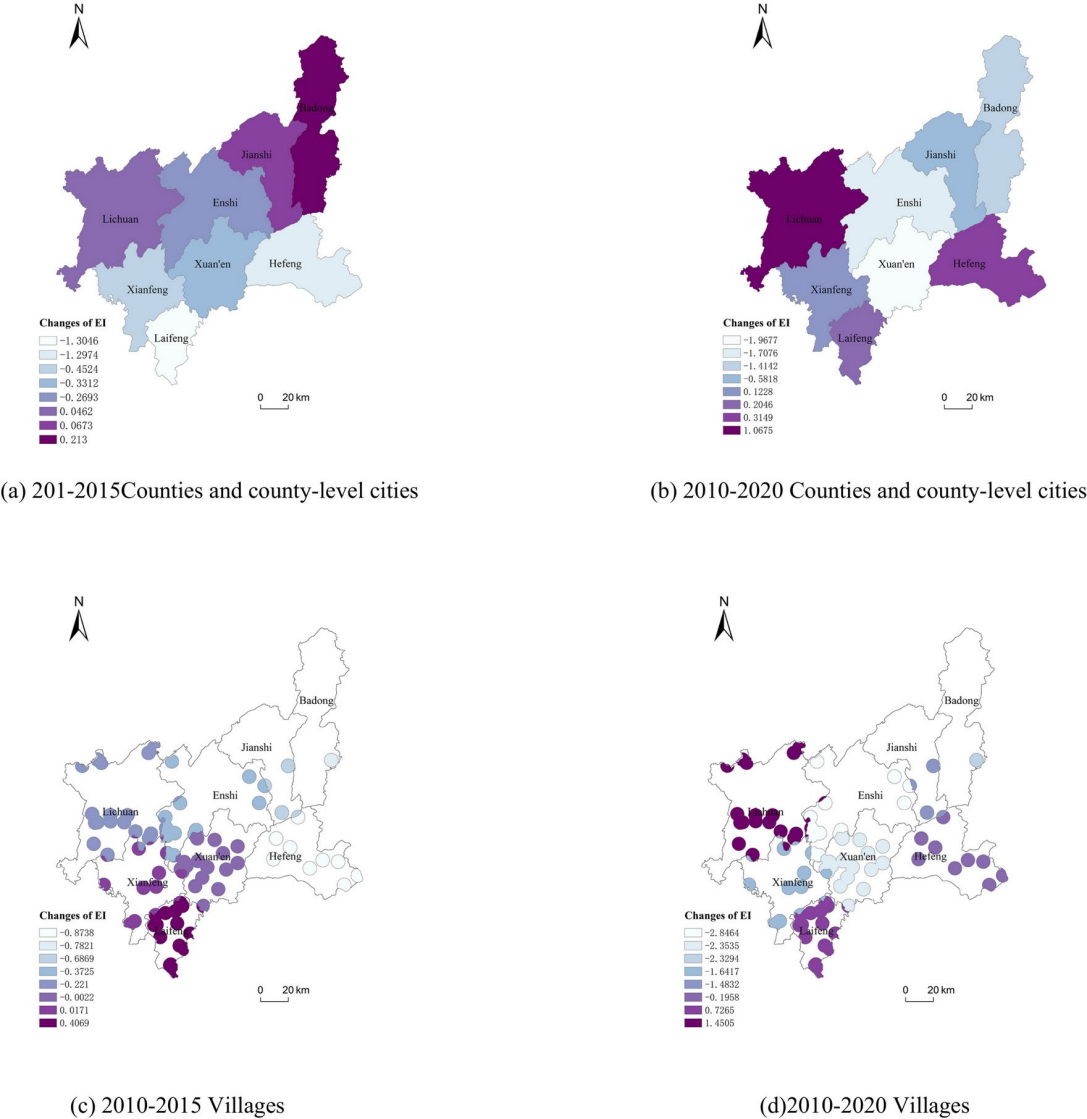
The changes of *EI* in Enshi Prefecture from 2010 to 2020.

The spatial distribution of *EI* changes in the counties and county-level cities of Enshi Prefecture and villages under their jurisdiction during the ten-year period is shown in Fig. 11, and it can be seen from Fig. 11b,d that the changes in ecological environmental conditions in the study area showed the most obvious changes in the central and western regions, and the changes in other regions were smaller. In terms of time series, for individual counties and county-level cities, the changes in ecological environmental conditions were both better and worse, and by analyzing the magnitude of the changes in *EI*, we know that the changes in the latter five years were basically greater than those in the first 5 years. Figure 11d shows that the obvious changes in the ecological environmental conditions of the villages in the counties and county-level cities were concentrated in the central region, in the regions of Enshi and Xuan’en.

The number of traditional villages in the central region of Enshi Prefecture was statistically higher, while the number of traditional villages in other regions was much lower. Correlation analysis of the number of traditional villages in each county and county-level city with the *EI* values of the villages under their jurisdiction showed that the change values of the ecological environmental conditions of the villages in each county and county-level city during the ten years had a weak linear positive correlation with the number of traditional villages in the region (the correlation coefficient was 0.518).

Overall, the ecological condition of the villages in the counties and county-level cities changed more significantly than that of the counties and county-level cities during the 10-year period. It can be seen that both the number of traditional villages and the number of years after the implementation of preservation policies for traditional villages had a certain impact on the ecological environmental status of the region. Comparing the distribution of *EI* in 2010, 2015 and 2020, it can be seen that the ecological environment preservation through the implementation of the traditional village preservation policy had a more significant effect on the ecological environment of the area around the villages, and the ecological environment condition of some areas continued to get better, while some areas got worse, and this change was more and more obvious with the passage of time.

To summarize, in the past 10 years, the ecological level of Enshi Prefecture still needed to be improved, but the ecological level of some areas had been improved. Both natural and human factors can affect the local ecological environment, and when protecting villages, we should combine the local ecological environment to formulate appropriate preservation programs.

## Discussion

Based on GIS and RS technologies and referring to the specification for evaluation of ecological environment conditions^33^, this paper took the counties and county-level cities of Enshi Tujia and Miao Autonomous Prefecture and the 5 km surrounding range around the traditional villages in the counties and county-level cities as the research objects, and constructed an evaluation model of ecological environment conditions by selecting four sub-indices of ecological environment conditions, and quantitatively analyzed the ecological environment conditions and their spatial–temporal change characteristics of the study area from 2010 to 2020. The characteristics of the ecological environment in the study area from 2010 to 2020 were quantitatively analyzed and the spatial and temporal changes of the ecological environment in the study area were quantitatively analyzed, and the differences between the overall ecological environment in the counties and municipalities and the ecological environment of the villages under their jurisdiction were revealed through comparative analysis, so as to explore the mechanism of the influence of the traditional village preservation policy on the ecological environment around the village.

During the period from 2010 to 2020, the vegetation coverage indices of all counties and county-level cities in Enshi Tujia and Miao Autonomous Prefecture and the villages under their jurisdiction showed a trend of increasing and then decreasing, the water network denseness index declined and then increased, and the land stress index increased, while the habitat quality index remained basically unchanged. The trends of the indices of the villages and the corresponding indices of the counties and county-level cities under their jurisdictions are basically consistent, but there is a big difference between them in terms of the values of the indices.

In 2010, 2015 and 2020, all counties and county-level cities, as well as traditional villages, were rated as “good” in terms of ecological condition. In 2020, compared with 2010, the *EI* value of villages changed more significantly than that of their counties and county-level cities, and the number of counties and county-level cities with “slight changes” was higher. Changes in the ecological condition index of villages and the number of villages were basically positively and linearly correlated.

The benefits of the implementation of the traditional village preservation policy are closely related to the local natural environment, and the natural conditions play an important role in the preservation of traditional villages. With the implementation of the traditional village preservation policy, in addition to the changes in soil erosion, water resources and land use in the above analysis, the ecological environment of the village was more and more obviously affected by human activities, and the impact of the traditional village preservation policy on the regional ecological environment was greater than that in the natural state.

The impact of the traditional village preservation policy on the ecological environment around the villages during the ten years was not always positive, and the *EI* values of the villages in some regions showed a decreasing trend, indicating that the human activities under the policy preservation had a negative impact on the ecological environment around the villages. This may be related to the local tourism economy, traditional villages may attract a large number of tourists to visit, increasing the pressure of local transportation, catering and accommodation. If not properly managed, commercialization and modernization brought about by tourism development may have certain impacts on and damage the natural environment.

Based on the conclusions of this paper, the following suggestions are made for the preservation policy of traditional villages in China. In the process of historical development, traditional villages can survive and develop, and the surrounding natural and humanistic environment is inseparable. The government and various departments should pay full attention to the village’s natural environment, spatial layout and other elements when formulating the preservation plan and program, so as to be able to play a certain degree to improve the role of the surrounding ecological environment conditions.

## Conclusion and outlook

The primary objective of this study is to quantitatively analyze the magnitude of impact that the implementation of traditional village conservation policies has had on the surrounding ecological environment. In the protection of traditional villages, protecting their natural landscape environment such as topography, rivers, lakes, and water systems is an important part. This article uses changes in the ecological environment of traditional villages to reflect the effectiveness of protecting their natural landscape environment, providing new ideas for understanding the impact and ecological effects of protecting traditional villages on the ecological environment. Specifically, from 2010 to 2020, the Enshi Tujia and Miao Autonomous Prefecture exhibited inconsistent trends in vegetation coverage, water network density, land stress, and habitat quality. For instance, fluctuations in vegetation coverage and water network density, along with a continuous rise in the land stress index, revealed the tension between economic development and ecological balance within the region. Efforts have been made by the government to strike a balance between these two aspects. Notably, the stability of the habitat quality index may be attributed to the effective implementation of ecological conservation policies, yet further analysis is required to determine its correlation with specific protective measures or natural recovery forces. Moreover, despite the general alignment in the ecological and environmental condition trends between villages and their respective county-level cities, discrepancies exist in their habitat quality index values. This suggests that traditional village conservation policies have exerted a certain degree of influence on the surrounding ecological environment. The study also confirmed that traditional village conservation policies have, to a certain extent, contributed to the improvement of the ecological environment. However, in some areas, the implementation of these policies has led to a decline in habitat quality, indicating that the protective measures may not have effectively curbed the negative impacts of human activities. Therefore, in future research and practice, it is imperative to more meticulously consider the comprehensive effects of policies, particularly in weighing the pros and cons between tourism development and environmental protection. The protection of traditional villages is not merely about preserving their physical form but, more importantly, ensuring the health and sustainability of their ecosystems. As the impact of human activities increases, a significant challenge we face is how to optimize policy design based on respect for natural laws and reduce adverse effects.

We employed GIS spatial analysis methods and remote sensing monitoring techniques to investigate changes in the natural ecological environment surrounding traditional villages before and after the implementation of protection policies. This approach revealed the positive or negative effects of policy interventions on vegetation, landforms, water resources, and biodiversity. Our study provides empirical data to support the optimization of village protection policies within the study area, aiding in achieving a balance between cultural heritage preservation and ecological environment protection. Additionally, it expands the scope of research on traditional village protection policies within the cultural and ecological domains in China, offering policymakers a more comprehensive empirical foundation. The quantitative analysis results presented in this paper enable a more scientific and precise evaluation of policy effectiveness, thus promoting a sustainable development model where cultural heritage conservation and the natural environment coexist harmoniously. This work provides a valuable reference for the implementation and enhancement of China’s cultural heritage protection policies in the future.

In light of the aforementioned findings, future research could be conducted in the following directions. Firstly, to delve into the specific factors contributing to the decline in habitat quality values in the study area, with particular focus on the mechanisms of tourism’s impact on the environment^36^. Secondly, to evaluate the long-term effects of traditional village conservation policies by collecting and analyzing environmental monitoring data from future villages to quantify the policies’ impact on the surrounding ecological environment and assess their effectiveness. Lastly, to employ higher-resolution time series data analysis methods to capture both short-term fluctuations and long-term trends in ecological changes.

## Data Availability

The datasets used and/or analysed during the current study available from the corresponding author on reasonable request.
